# Geraniol inhibits biofilm formation of methicillin-resistant *Staphylococcus aureus* and increase the therapeutic effect of vancomycin *in vivo*

**DOI:** 10.3389/fmicb.2022.960728

**Published:** 2022-09-06

**Authors:** Kexin Gu, Ping Ouyang, Yuxin Hong, Yuyun Dai, Ting Tang, Changliang He, Gang Shu, Xiaoxia Liang, Huaqiao Tang, Ling Zhu, Zhiwen Xu, Lizi Yin

**Affiliations:** College of Veterinary Medicine, Sichuan Agriculture University, Chengdu, China

**Keywords:** geraniol, MRSA, biofilm, PIA, eDNA, sarA, staphyloxanthin, implant model

## Abstract

Methicillin-resistant *Staphylococcus aureus* (MRSA) is among the common drug resistant bacteria, which has gained worldwide attention due to its high drug resistance and infection rates. Biofilms produced by *S. aureus* are known to increase antibiotic resistance, making the treatment of *S. aureus* infections even more challenging. Hence, inhibition of biofilm formation has become an alternative strategy for controlling persistent infections. In this study, we evaluated the efficacy of geraniol as a treatment for MRSA biofilm infection. The results of crystal violet staining indicated that 256 μg/mL concentration of geraniol inhibited USA300 biofilm formation by 86.13% and removed mature biofilms by 49.87%. Geraniol exerted its anti-biofilm effect by influencing the major components of the MRSA biofilm structure. We found that geraniol inhibited the synthesis of major virulence factors, including staphyloxanthin and autolysins. The colony count revealed that geraniol inhibited staphyloxanthin and sensitized USA300 cells to hydrogen peroxide. Interestingly, geraniol not only reduced the release of extracellular nucleic acids (eDNA) but also inhibited cell autolysis. Real-time polymerase chain reaction data revealed the downregulation of genes involved in biofilm formation, which verified the results of the phenotypic analysis. Geraniol increased the effect of vancomycin in eliminating USA300 biofilms in a mouse infection model. Our findings revealed that geraniol effectively inhibits biofilm formation *in vitro*. Furthermore, in combination with vancomycin, geraniol can reduce the biofilm adhesion to the implant in mice. This suggests the potential of geraniol as an anti-MRSA biofilm drug and can provide a solution for the clinical treatment of biofilm infection.

## Introduction

*Staphylococcus aureus* is a common gram-positive bacterium that remains the main cause of healthcare-associated infections (Tenover et al., 2006). Due to the unreasonable use of antibiotics, many antibiotic-resistant strains have been found (Catalano et al., 2022). Methicillin-resistant *Staphylococcus aureus* (MRSA) is a well-known drug-resistant bacterium. In adults, MRSA is the main pathogen that causes bacteremia, pneumonia, and endocarditis (Turner et al., 2019). To date, vancomycin has always been the drug of choice for the treatment of MRSA infections and the last line of defense against MRSA. Unfortunately, vancomycin-resistant strains have been isolated since the beginning of the twenty-first century, worsening the current situation (Cong et al., 2020). Therefore, finding novel drugs that exhibit antibacterial activity but do not cause antibiotic resistance has attracted increasing attention worldwide.

Numerous infections caused by MRSA are closely related to biofilms. The biofilm structure prevents the complete eradication of pathogenic bacteria using antibiotics. MRSA biofilms mainly comprise polysaccharide intercellular adhesin (PIA), extracellular nucleic acids (eDNA), and proteins called extracellular polymeric substances (EPS). EPS provide protection against bacteria wrapped in biofilms, including evading the immune system and avoiding exposure to antibiotics (Sharma et al., 2019). Biofilm formation is an extremely complex and closely regulated process. SarA, a well-known *S. aureus* regulatory system, regulates the expression of many important virulence factors and induces *S. aureus* to produce biofilms, mainly by inhibiting the secretion of proteases (Trotonda et al., 2005; Abdelhady et al., 2014). It has been reported that not only is the biofilm-forming ability of *sarA* mutants seriously damaged but it also leads to changes in the expression levels of at least 120 genes (Mauro et al., 2016; Oriol et al., 2021). Numerous natural compounds, including thymol, carvacrol, and hesperidin, have been found to combat MRSA biofilms by targeting SarA (Selvaraj et al., 2020; Yuan et al., 2020; Vijayakumar et al., 2022). Taken together, these results indicated that *sarA* is a promising anti-biofilm target.

Geraniol is a terpenoid compound that is well known as the main component of many essential oils (Xu et al., 2022). Geraniol is found mainly in aromatic herbaceous plants and is cost-effective and easy to produce (Prasad and Muralidhara, 2017; Younis et al., 2021). In previous studies, geraniol was found to have anti-neurotoxicity properties (Lin et al., 2021), inhibit gastric cancer cell proliferation (Yang et al., 2021), and relieve arthritis (Wu et al., 2020). In recent years, geraniol has been proven to have significant antibacterial activity against gram-negative bacteria, such as *Escherichia coli* and *Salmonella* Typhimurium, (Friedman et al., 2002), and *Streptococcus pneumoniae* and *S. aureus* (Inouye et al., 2001). It has also been reported that geraniol can inhibit the growth of MRSA and relieve the symptoms of systemic infection caused by MRSA in mice (Lin et al., 2021). Furthermore, geraniol inhibits biofilm production by a variety of bacteria (Gupta et al., 2021; Kwiatkowski et al., 2022; Yu et al., 2022). However, the inhibitory effect and mechanism of action of geraniol on MRSA biofilms have rarely been reported. This study shows that geraniol inhibits the formation of MRSA biofilms by regulating the expression of the sarA system regulatory genes, genes related to virulence factors, and other genes that regulate biofilm formation. Vancomycin is clinically used in the treatment of MRSA infections. However, it causes nephrotoxicity and large doses may cause bacterial drug resistance. In an aim to establish the efficacy of geraniol in combination with vancomycin and reduce the dose of vancomycin, we conducted the current study.

## Results

### Geraniol has an effect on the growth of USA300

**FIGURE 1:**
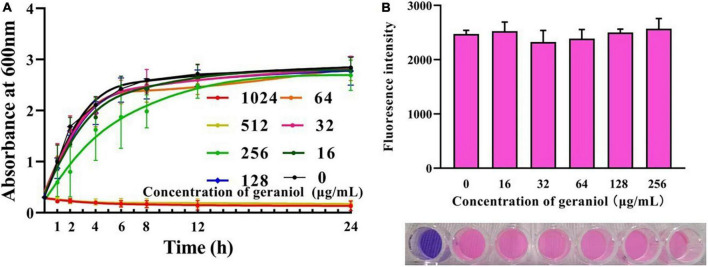
Effects of geraniol at different concentrations on the activity of USA300 assessed by the **(A)** growth curve assay and **(B)** Alamar blue analysis.

Geraniol exhibited antibacterial activity against USA300 with minimum inhibitory concentration (MIC) and minimum bactericidal concentration values of 512 and 1,024 μg/mL, respectively. The MIC optical density values at 600 nm (OD_600 nm_) are shown in Supplementary Table 1. The growth curve (Figure 1A and Supplementary Table 2) showed that geraniol did not inhibit the growth of USA300 at concentrations of 0, 16, 32, 64, 128, and 256 μg/mL.

### Biofilm formation inhibition effects of geraniol against USA300 and several other *Staphylococcus aureus* strains

**FIGURE 2:**
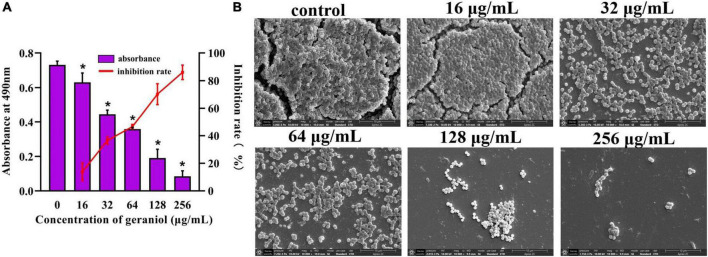
Effect of geraniol with sub-minimum inhibitory concentrations on the formation of USA300 biofilms assessed by **(A)** crystal violet staining and **(B)** scanning electron microscopy images at 10,000 × magnification. *p < 0.05 compared with the 0 μg/mL group.

Biofilm experiments were performed at sub-inhibitory concentrations of geraniol. The results of the Alamar Blue test (Figure 1B) showed that there was a negligible difference in the fluorescence intensity between the drug treatment group and untreated group, indicating that the activity of USA300 was hardly affected by the sub-MICs of geraniol. Geraniol significantly inhibited biofilm formation induced by USA300. When the concentration of geraniol was 128 and 256 μg/mL, biofilm formation decreased by 70.29 ± 7.58% and 86.13 ± 5.22%, respectively (Figure 2A). Figure 2B shows that the slides containing samples from the untreated group were densely covered with cells. However, as the geraniol concentration increased, the number of cells decreased, and PIAs had exuded. Only a small number of unaggregated cells was observed in the group treated with 256 μg/mL geraniol. These results indicated that the sub-MICs of geraniol (16, 32, 64, 128, and 256 μg/mL) significantly inhibited USA300 biofilm formation in a dose-dependent manner. In addition to USA300, the effect of geraniol on biofilm formation of several other *S. aureus* strains was also evaluated (Supplementary Figure 1). The biofilm inhibition rate of geraniol on these strains was more than 50%. The MIC of geraniol on these strains is shown in Supplementary Table 3.

### Geraniol removed preformed USA300 biofilms and several other *Staphylococcus aureus* strains

**FIGURE 3:**
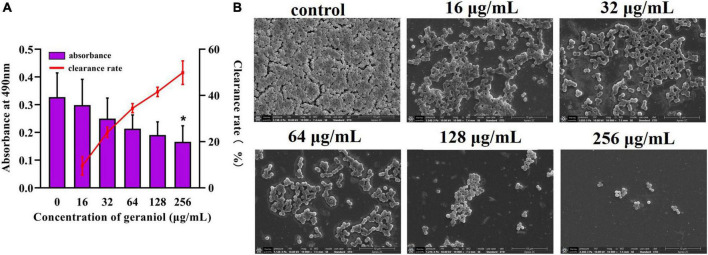
Effect of geraniol with sub-minimum inhibitory concentrations removing on preformed biofilms of USA300. **(A)** Crystal violet quantification and **(B)** scanning electron microscopy images at 10,000 × magnification. *p < 0.05 compared with the 0 μg/mL group.

The biofilm removal assay revealed that geraniol, at concentrations of 256 μg/mL, effectively disrupted preformed biofilms, decreasing them by 49.87 ± 5.11% (Figure 3A). The scanning electron microscopy (SEM) observations (Figure 3B) showed that the slides without geraniol treatment were completely covered by bacteria and the bacterial cells were closely arranged like pavers blocks. As the concentration increased, bacteria gradually decreased, and the distribution of bacterial cells became scattered. Geraniol destroyed the structure of the biofilm. Geraniol also had different scavenging effects on preformed biofilms of other *S. aureus* strains. However, the effect on 26FS31, YFC18 and 2ZG3 clinical strains was not significant (Supplementary Figure 2).

### Qualitative analysis of polysaccharide intercellular adhesin production

**FIGURE 4:**
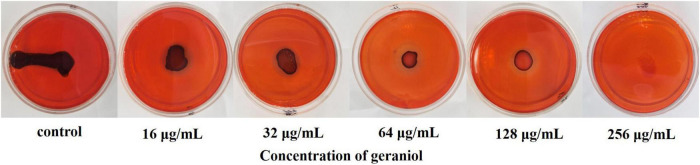
Qualitative analysis of the polysaccharide intercellular adhesin of USA300 upon geraniol treatment.

In the Congo Red (CR) plate, the production of PIA was indicated by the number of black colonies, with the PIA-positive bacterial strain being completely black. The results presented in Figure 4 show that as the geraniol concentration increased, the PIA synthesis gradually inhibited (observed as reduced color intensity of the black colonies). This suggests that geraniol inhibited USA300 biofilms by reducing the synthesis of PIA.

### Qualitative analysis of extracellular DNA release

**FIGURE 5:**
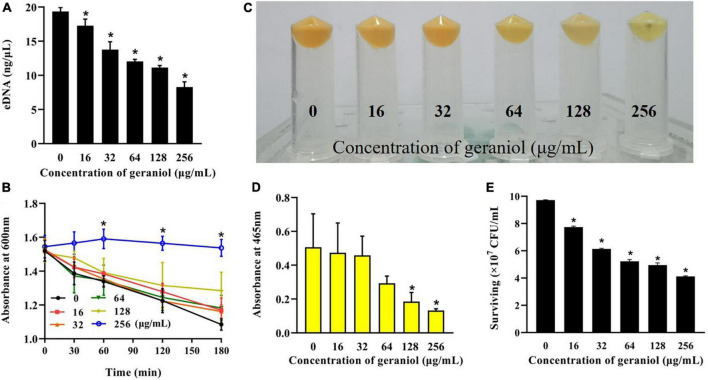
Inhibitory effects of geraniol on the release of extracellular nucleic acids and staphyloxanthin biosynthesis of USA300. **(A)** eDNA release, **(B)** autolysis, **(C)** cell color, **(D)** qualitative analysis of staphyloxanthin, and **(E)** effect of geraniol treatment on the survival of USA300 in H_2_O_2_. *p < 0.05 compared with the 0 μg/mL group.

The amount of eDNA released by USA300 cells was evaluated in the absence and presence of geraniol. As shown in Figure 5A, at low concentrations of geraniol, the release of eDNA from MRSA biofilms was significantly inhibited. Compared to that in the untreated group, eDNA release was decayed by 42.50 ± 1.57% and 57.10 ± 4.59% after treatment with 128 and 256 mg/mL geraniol, respectively.

### Cell autolysis assay

Autolysis of USA300 with 128 μg/mL geraniol was negligible in the first 30 min, whereas autolysis of USA300 treated with 256 μg/mL geraniol was effectively inhibited (Figure 5B).

### Geraniol inhibited the synthesis of staphyloxanthin and sensitized USA300 to H_2_O_2_

In geraniol-treated cells, the color of the bacteria turned pale (Figure 5C), and staphyloxanthin production (Figure 5D) was inhibited up to 70.70 ± 12.34%, with this inhibition being concentration-dependent. As shown in Figure 5E, the sensitivity of USA300 to H_2_O_2_ increased significantly in the 256 μg/mL geraniol-treated group (the number of live bacteria was 4.12 × 10^7^ CFU/mL) compared with the untreated group (the number of live bacteria was 9.72 × 10^7^ CFU/mL).

### Effect of geraniol on the expression of genes

**FIGURE 6:**
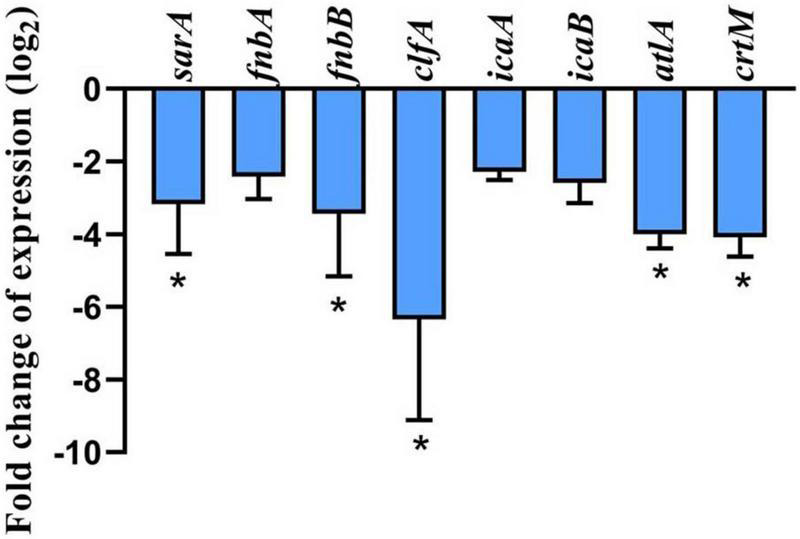
Effect of geraniol at 256 μg/mL concentration (half of the minimum inhibitory concentration) on the expression of genes involved in biofilm formation and related virulence factors of USA300. *p < 0.05 compared with the 0 μg/mL group.

The results of the quantitative polymerase chain reaction (qPCR) (Figure 6) showed the effect of 256 μg/mL geraniol on the genes involved in biofilm formation and virulence factor production in USA300. Geraniol downregulated the expression of *sarA*, *fnbA*, *fnbB*, *clfA*, *icaA*, *icaB*, *atlA*, and *crtM*.

### Vancomycin combined with geraniol reduced intraperitoneal foreign-body biofilm infection caused by methicillin-resistant *Staphylococcus aureus* in mice

#### Vancomycin combination with geraniol removed preformed biofilms

**FIGURE 7:**
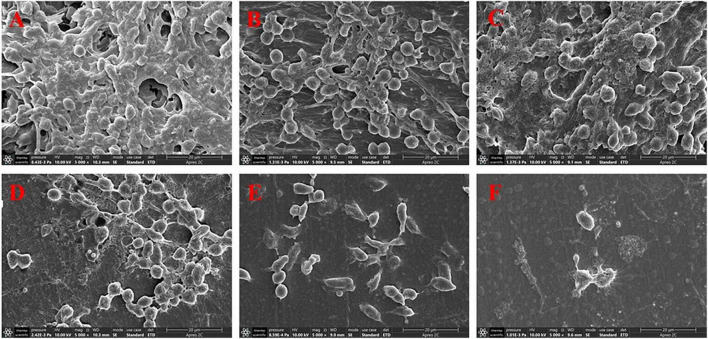
Scanning electron microscopy images at 5,000 × magnification of biofilms in peritoneal implants from mice. The doses of the drug were **(A)** physiological saline, **(B)** 40 mg/kg geraniol, **(C)** 40 mg/kg vancomycin, **(D)** 10 mg/kg geraniol + 40 mg/kg vancomycin, **(E)** 20 mg/kg geraniol + 40 mg/kg vancomycin, and **(F)** 40 mg/kg geraniol + 40 mg/kg vancomycin.

The microscopic analysis showed that biofilms were removed to varying degrees. As shown in Figure 7, in the physiological saline group, the implant was tightly covered with dense biofilm bacteria, and many bacteria adhered to each other in clumps. A reticular structure was observed between the bacteria. In the high-dose combination group, the preformed biofilms were almost completely removed, and only a few bacteria were observed. A completely disrupted biofilm architecture was observed, with reticula-connecting bacteria that had already been destroyed.

#### Vancomycin combined with geraniol reduced bacterial adhesion

**FIGURE 8:**
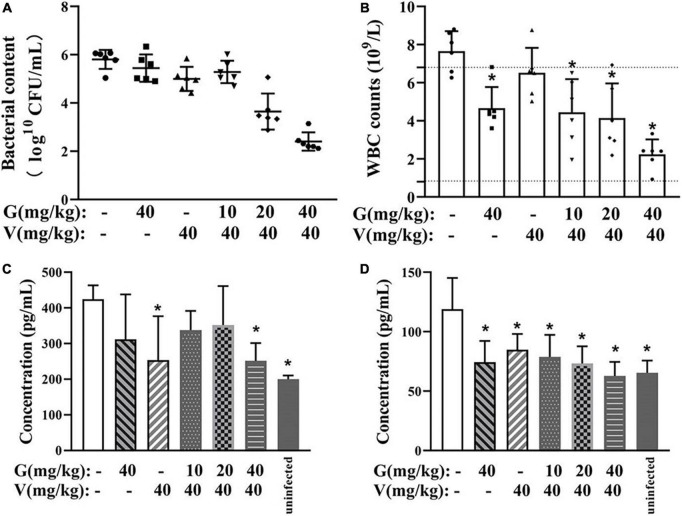
Therapeutic effect of combinations of vancomycin and/or geraniol on the mice infection model. **(A)** Bacterial count in implants, **(B)** white blood cell counts in mice, serum **(C)** TNF-α and **(D)** IL-6 levels in model mice after treatment. *p < 0.05 compared with the 0 μg/mL group.

The bacterial burden on the implants was calculated using colony counting. The implants treated with a combination of geraniol (40 mg/kg) and vancomycin (40 mg/kg), which showed the most significant effect, exhibited minimal bacterial adhesion, and bacterial counts (Figure 8A) were lower than those observed in the physiological saline group (99.94% ± 0.06%).

#### Vancomycin combined with geraniol reduced the inflammatory responses in mice

In all treatment groups, the white blood cell (WBC) count decreased to a normal level, and the levels of TNF-α and IL-6 were inhibited. Particularly, compared with the physiological saline group, the WBC count in the high-dose group (geraniol 40 mg/kg + vancomycin 40 mg/kg) decreased by 70.11% ± 10.70% (Figure 8B), and the levels of TNF-α and IL-6 were significantly lower by 46.33 ± 7.45% and 41.01 ± 7.90%, respectively (Figures 8C,D).

## Discussion

The limitation of antibiotic resistance caused by drug-resistant bacteria, such as MRSA and vancomycin-resistant *S. aureus*, has raised widespread concern worldwide. MRSA can cause chronic nosocomial infections and adhere to medical devices, especially implantable medical devices, by forming biofilms. Therefore, the demand for compound-targeted biofilms has increased in recent years. Anti-biofilm is a promising strategy for the treatment of MRSA-induced infections (Roy et al., 2018). Several natural compounds, such as limonene, andrographolide sulfonate, and luteolin, have been found to inhibit biofilm formation (Zhang et al., 2021; Gambino et al., 2022; Yuan et al., 2022). In this study, the growth curves and Alamar Blue assay indicated that the tested concentration of geraniol did not affect the activity of bacteria. Indicating that the drug concentration will not have an effect on subsequent biofilm formation. This property is not easy to cause drug resistance in bacteria (Roy et al., 2018). Moreover, we found that geraniol has a transcendent activity of inhibiting MRSA biofilm formation and has a superior mature biofilms elimination effect. Furthermore, to evaluate the inhibitory activity of geraniol on biofilm as comprehensively as possible, we observed the effect of geraniol on biofilm formation of methicillin-sensitive *S. aureus* (MSSA) and several other MRSA strains *in vitro*. Geraniol also has an apparent biofilm formation effect on these strains, which indicates that geraniol has a wide range of effects.

The PIA produced by *S. aureus* is an important contributor to biofilm formation. PIA contributes to stabilizing the interaction between bacterial cells and the adhesion of *S. aureus* to medical devices (Schilcher and Horswill, 2020). Nguyen et al. (2020) reported that wild-type strains cause more severe infections and show higher survival rates than PIA mutant strains in infected mouse models. Overall, determining the effect of geraniol on PIA production is important for exploring the potential mechanisms of geraniol. Our findings indicated that geraniol reduced PIA production in USA300 cells, as qualitatively evaluated using the CR plate method. PIA biosynthesis is regulated by the icaADBC operon. *icaA* encodes the main PIA synthesis, *icaB* is critical to the PIA adhesion function because it introduces a positive charge into the PIA via deacetylation, allowing the bacteria to adhere stably to surfaces (Nuryastuti and Krom, 2017; Lerch et al., 2019; Schilcher and Horswill, 2020). However, *icaA* cannot be expressed without *sarA*, since the promoter of the ica operon requires binding to *sarA* (Tamber and Cheung, 2009). *sarA* is an anti-biofilm target that has been discovered recently. It was reported that for individual strains, *ica* is not necessary for biofilm formation (Trotonda et al., 2005; Dengler Haunreiter et al., 2019). However, in *ica*-independent strains, *sarA* regulates biofilm formation in other ways. For instance, *sarA* positively regulates *fnbA*, *fnbB*, and *clfA* (determinants of *S. aureus* surface adhesins) (Pantrangi et al., 2015; Swarupa et al., 2018). Therefore, we selected several core genes for this study and found that they were significantly downregulated under the influence of geraniol. This is in agreement with phenotypic experiments (Zhang et al., 2021; Yehia et al., 2022).

In addition to PIA, eDNA is another major constituent of MRSA biofilms. The addition of DNase I (an enzyme that degrades eDNA) significantly reduces the formation of biofilms and facilitates its eradication (Nagasawa et al., 2017; Soler-Arango et al., 2019). The amount of eDNA released in this study (Figure 5A) supported the biofilm results, that is, geraniol inhibited the formation of biofilms and decreased the release of eDNA. Similar to apoptosis, *S. aureus* undergoes a cleavage process regulated by conserved genes, which is called autolysis (Rice et al., 2007). Autolysin is encoded by *atlA* and is involved in bacterial cell wall homeostasis and peptidoglycan conversion. Previous studies have shown that the release of eDNA by *S. aureus* is mediated by autolysis and biofilm formation (Rice et al., 2007; Houston et al., 2011). When the expression level of *atlA* was downregulated, the biofilm formation ability of *S. aureus* decreased sharply (Biswas et al., 2006). Therefore, we studied the autolysis of USA300 cells treated with geraniol, and found that geraniol significantly inhibited cell autolysis. This is consistent with the results of previous studies (Selvaraj et al., 2019; Valliammai et al., 2019) and suggests that geraniol reduces the release of eDNA by inhibiting cell autolysis. The gene expression level also confirmed that geraniol downregulated *atlA* gene. Hence, geraniol affected the USA300 biofilm architecture by modulating the expression of *atlA*.

Notably, geraniol changed the color of USA300 in this experiment. We speculate that this was associated with staphyloxanthin biosynthesis. Staphyloxanthin is the main component of the bacterial pigment that provides *S. aureus* its unique yellow or orange appearance. Staphyloxanthin protects *S. aureus* from escaping the immune system and from the bactericidal effect of oxides, which promotes bacterial survival (Liu and Nizet, 2009). This is due to the fact that the alternate bonds in the staphyloxanthin structure can absorb excess energy from reactive oxygen species (El-Agamey et al., 2004). Previous studies have shown that mutants encoding staphyloxanthin synthase CrtM not only cause *S. aureus* to lose its yellow appearance but are also more likely to be killed by peroxides in whole blood (Clauditz et al., 2006). The results of the lethal analysis and qPCR showed that geraniol could improve the sensitivity of USA300 to H_2_O_2_ and downregulate *crtM* gene expression, further verifying the results of phenotypic experiments. These results suggest that geraniol may be beneficial in the clinical treatment of MRSA.

Despite many preventive measures, medical device infections caused by biofilms still occur often, particularly implanted device infections. It is especially important to evaluate the biofilm scavenging activity of drugs *in vivo*. Therefore, we explored the therapeutic effects of geraniol combined with vancomycin on foreign body infections in mice. After 24 h of treatment, the pathological damage in mice was significantly alleviated. The therapeutic effect was the most obvious in the high-dose group. Neither geraniol nor vancomycin effectively killed bacteria that adhered to the surface. However, the number of bacteria significantly decreased with the addition of geraniol. Based on these results, we presumed that geraniol could effectively destroy the biofilm structure in mice and increase the effect of vancomycin. Infection with *S. aureus* causes an increase in WBCs and a large release of proinflammatory factors. In this study, the WBC count and the release of TNF-α and IL-6 in the dosing group were downregulated. Due to the high bacteriostatic concentration of geraniol (MIC = 512 μg/mL), we speculate that the anti-inflammatory effect of geraniol alleviates inflammatory symptoms in mice (Li et al., 2018; Toiu et al., 2019; Yuan et al., 2020). Owing to vancomycin’s non-negligible toxicity, the dosage of vancomycin should not be increased in clinical practice. The *in vivo* results indicated that the combination of geraniol and vancomycin could effectively treat biofilm infection, which is critical for the treatment of MRSA biofilms.

In summary, geraniol not only effectively prevented MRSA biofilm formation but also removed mature MRSA biofilms. Geraniol inhibited the secretion of PIA and released eDNA, mainly by inhibiting the gene expression of *sarA* and *atlA*. Furthermore, geraniol reduced staphyloxanthin production by downregulating *crtM*, thereby increasing the sensitivity of MRSA to peroxides. In this study, the ability of geraniol in combination with vancomycin to remove biofilms *in vivo* was also evaluated. Although geraniol alone did not significantly remove the biofilm from the implant in mice, it is speculated that geraniol improved the therapeutic effect of vancomycin by destroying the structure of the biofilms, relieving inflammatory symptoms. In conclusion, geraniol is a potential drug for treating MRSA biofilms. However, the pharmacodynamics, pharmacokinetics, and toxicity of geraniol require further exploration.

## Materials and methods

### Bacterial strain and drug reagent

The MRSA strain USA300 (ATCC^®^ BAA-1717™) [obtained from the American Type Culture Collection (ATCC)] was used in the current study and was cultivated in brain heart infusion (BHI) broth (Hopebio, Qingdao, China). The bacteria were cultured in an air bath constant temperature oscillator (BS-2F, Ningbo Jingda Formal Equipment Co., Ltd., Jintan, China) at a temperature of 37°C and a rotational speed of 150–200 rpm. Geraniol (>98% HPLC purity; CAS No. 106-24-1) was purchased from Shanghai McLean Biochemical Technology Co., Ltd. (Shanghai, China) and dissolved in dimethyl sulfoxide to obtain a stock solution. Vancomycin was purchased from Beijing Solarbio Science and Technology Co., Ltd. MSSA (ATCC^®^ 25923, ATCC^®^ 29213) were obtained from the ATCC. MRSA (ATCC^®^ 43300, PHY6, 26FS18, C2Y, 2ZG3, YFC31) were presented by Professor Yanhua Li, School of Animal Medicine, Northeast Agricultural University.

### Ethics statement

All animal studies were performed in accordance with the approved experimental practices and standards of the Animal Ethics Committee of Sichuan Agricultural University (Chengdu, China), and the experimental protocols were approved and conducted under the supervision of the Animal Care Committee (permitnumberDKY-B2020203001; date of approval: June 11, 2021).

### Effect of geraniol on the activity of USA300

#### Susceptibility testing and growth curve assay

The MIC of geraniol for *S. aureus* strains was determined using the double dilution method according to the Clinical and Laboratory Standards Institute (Arendrup et al., 2017). For USA300, after the bacteria were static cultured at 37°C for 18 h, the OD_600 *nm*_ of each bacteria group was determined. In the determination of the growth curve according to Yuan’s experimental method (Yuan et al., 2019), the OD_600 *nm*_ was measured at different time points, which indicated the growth of USA300 (at 37°C and 200 rpm shaking speed) co-cultured with different concentrations (0, 16, 32, 64, 128, 256, 512, and 1,024 μg/mL) of geraniol. An appropriate amount of DMSO was used instead of geraniol as the control group in all experiments.

#### Alamar blue assay

Cell viability was evaluated using the Alamar Blue (Solaibao, Beijing, China) assay according to Khan’s experimental method (Khan et al., 2022). Briefly, Alamar Blue reagent was mixed with USA300 cells treated with sub-MIC (0, 16, 32, 64, 128, and 256 μg/mL) of geraniol. Samples were incubated in opaque 96-well plates (BKMAM BIOTECHNOLOGY Co., Ltd., Hunan, China) for 30 min at 37°C, before the fluorescence intensity was determined.

### Effect on biofilm formation

#### The effect of geraniol on the formation of methicillin-resistant *Staphylococcus aureus* biofilms

Referring to the method of Peng et al. (2020), MSSA and MRSA strains cells (treated with sub-MICs of geraniol or appropriate concentration of DMSO) were cultured in 96-well plates (BKMAM BIOTECHNOLOGY Co., Ltd., Hunan, China) under anaerobic condition at 37°C for 18 h, and the mature biofilm was gently washed with non-heat source phosphate-buffered saline (PBS) to remove planktonic bacteria. Then the cultures were fixed by formaldehyde solution for more than 8 h and mature biofilms were stained with crystal violet for 2 h. Acetic acid (290 μL/well) was added to each well to dissolve the adhered dye, and the OD_490 *nm*_ was determined. SEM was used to observe morphological and structural changes in biofilms. The biofilms were cultured on slides (2 × 2 cm) using the same method as that used by Yuan et al. (2020). Finally, the biofilms were visualized using SEM (Apreo 2; Thermo Fisher Scientific Inc., Waltham, MA, United States).

#### Biofilm removal assay

Preformed biofilms were treated with sub-MICs of geraniol or appropriate concentration of DMSO (diluted with PBS) for 24 h at 37°C. Then, similar to the steps in the previous experiment, the supernatant was removed and the planktonic bacteria were gently washed out with non-heat source PBS once. After crystal violet staining, the OD_490 *nm*_ was determined. SEM samples were prepared using the same method as previously described (Selvaraj et al., 2019).

#### Polysaccharide intercellular adhesin analysis

To determine the effect of geraniol PIA production, the overnight (12 h) cultured USA300 suspension was inoculated on CR agar plates containing sub-MIC geraniol, and colony color was observed after incubation at 37°C for 24 h (Freeman et al., 1989).

#### Extraction of extracellular DNA

USA300 was diluted to 10^7^ CFU/mL, and fresh BHI broth (Hopebio) with sub-MICs of geraniol was added to a 6-well plate (NEST Biotechnology Co., Ltd., Zhejiang, China), co-cultured for 18 h to construct a mature biofilm, and extracted according to the Rice method (Rice et al., 2007). Briefly, the mature biofilm was re-suspended by TEN buffer and the supernatant was absorbed for centrifugation (4°C, 18,000 rpm, 5 min). The supernatant was mixed with TE buffer and mixed solution of organic reagent (phenol, chloroform, and isoamyl alcohol) and extracted. After the extract was centrifuged (4°C, 12,000 rpm, 10 min), the supernatant was re-extracted by chloroform and isoamyl alcohol. Finally, eDNA release was detected using an ultra-micro spectrophotometer (NanoDrop One, Thermo Fisher Scientific Inc., Waltham, MA, United States).

#### Autolysis analysis

USA300 cells were cultured overnight (12 h) in the presence of sub-MICs of geraniol. After washing thrice with PBS, the cells were resuspended in PBS containing 0.02% Triton Xmur100 (Solaibao, Beijing, China). These cells were cultured at 37°C and the OD_600 *nm*_ value of the bacterial suspension was determined every 30 min (Valliammai et al., 2019).

#### Pigment production assay

Overnight (12 h) cultured USA300 was added to BHI broth containing sub-MICs of geraniol at 37°C and 180 rpm shaking speed for 24 h. Suspended cells were collected and extracted with methanol for more than 8 h. Thereafter, the supernatant was collected, and the OD_465 *nm*_ value was determined (Yuan et al., 2020).

#### H_2_O_2_ killing assay

USA300 cells treated with sub-MICs of geraniol were added to PBS with H_2_O_2_ (1 mM) and incubated at 37°C for 1 h. The cells were then washed twice with PBS, diluted, evenly coated on BHI agar plates (Hopebio, Qingdao, China), cultured at 37°C for 24 h, and the colony count was performed (Valliammai et al., 2019).

#### Quantitative real-time PCR

Total RNA was extracted from USA300 cells grown in BHI broth to the late-logarithmic phase in the absence and presence of geraniol (256 μg/mL) using an RNA kit (Tianmo Biotech, Beijing, China) and converted to cDNA using 5X RT Mix (Beijing Biomed Gene Technology Co., Ltd., China). PCR was performed in a 20 μL reaction volume containing BlasTaq™ 2XqPCR MasterMix (abm, Vancouver, Canada) according to the manufacturer’s instructions. Real-time PCR was performed on a CFX Connect™ Real-Time PCR System (Bio-Rad Laboratories, Hercules, CA, United States) using specific primers (Table 1) designed using Primer 5.0. (PREMIER Biosoft, Canada) The levels of the target transcripts were calculated relative to those of 16S rRNA (housekeeping gene) using the 2^–ΔΔCt^ method (Schmittgen and Livak, 2008).

**TABLE 1:** Primer sequence.

Primer	Sequence (5′→3′)
16s-f	GCTGCCCTTTGTATTGTC
16s-r	AGATGTTGGGTTAAGTCCC
sarA-f	TTGTTTTCGCTGATGTAT
sarA-r	CAATGGTCACTTATGCTG
fnbA-f	ATCAGCAGATGTAGCGGAAG
fnbA-r	TTTAGTACCGCTCGTTGTCC
fnbB-f	AAGAAGCACCGAAAACTGTG
fnbB-r	TCTCTGCAACTGCTGTAACG
clfA-f	ATTGGCGTGGCTTCAGTGCT
clfA-r	CGTTTCTTCCGTAGTTGCATTTG
icaA-f	TTTCGGGTGTCTTCACTCTAT
icaA-r	CGTAGTAATACTTCGTGTCCC
icaB-f	ATGGTCAAGCCCAGACAGAG
icaB-r	AGTATTTTCAATGTTTAAAGCA
atlA-f	TGTCGAAGTATTTGCCGACTTCGC
atlA-r	TGGAATCCTGCACATCCAGGAAC
crtM-f	ATCCAGAACCACCCGTTTTT
crtM-r	GCGATGAAGGTATTGGCATT

### Animals

#### Establishment of a murine intraperitoneal foreign-body biofilm infection model in murine

The murine model was constructed according to the method by Yuan et al. (2020). Briefly, a USA300 biofilm was grown on the implants (a 1 mm in length medical PVC tubes, Yongkang, Shandong, China). Then, in the sterile environment, the mice were anesthetized with pentobarbital (40 mg/kg), a small incision was made in the left groin, the implants were placed carefully, and a professional sutured the incision. The process of establishing the mouse model is shown in Supplementary Figure 3. Finally, the mice were randomly divided into four groups with eight mice in each group, and the mice were injected with 0.1 mL of the drugs (40 mg/kg geraniol, 40 mg/kg vancomycin, 10 mg/kg geraniol + 40 mg/kg vancomycin, 20 mg/kg geraniol + 40 mg/kg vancomycin, 40 mg/kg geraniol + 40 mg/kg vancomycin, and physiological saline). All drugs were injected intraperitoneally twice daily (24 h) with a 12 h interval.

#### Colony counting

The implants were removed from the mouse and gently rinsed with non-heat source PBS to remove the attached impurities. Thereafter, the colonies on the implants were counted (Tursi et al., 2020).

#### Scanning electron microscopy

The implants were removed from the mice and fixed with 2.5% glutaraldehyde (Solaibao, Beijing, China) for 24 h; then the SEM (Apreo 2; Thermo Fisher Scientific Inc., Waltham, MA, United States) was used to observe the morphology and structure of the biofilms (Inoue et al., 2017).

#### White blood cell counts

At 12 h after the second administration, the blood of the mice was collected, and a blood cell analyzer (BC-5120; Mindray, Shenzhen, China) was used to count the WBCs.

#### Enzyme-linked immunosorbent assay

Blood was collected from the mice, and the serum was prepared according to the instructions of the enzyme-linked immunosorbent assay (ELISA) kit (Jiangsu Meimian industrial Co., Ltd., Yancheng, China) for ELISA.

### Statistical analysis

All *in vitro* experiments were performed in triplicate, and the values are presented as mean ± standard deviation. Two-tailed Student’s *t*-tests and analysis of variance were used to analyze statistically significant differences. GraphPad Prism 8 software was used for the analysis. Differences with *p*-values less than 0.05 were considered statistically significant.

## Data availability statement

The raw data supporting the conclusions of this article will be made available by the authors, without undue reservation.

## Ethics statement

The animal study was reviewed and approved by the Animal Ethics Committee of Sichuan Agricultural University (Chengdu, China).

## Author contributions

LY and ZX conceived and designed the experiments. KG, YH, YD, CH, XL, GS, and TT performed the experiments. TT and LZ contributed to preparing the reagents, materials, and analysis tools. KG and OP wrote the manuscript. All authors have read and agreed to the published.

## Supplementary material

Supplementary Figure 1 Inhibition rate of geraniol with sub-minimum inhibitory concentrations on the formation of MSSA standard strains **(A)** ATCC 29213, **(B)** ATCC 25923 and MRSA strains **(C)** ATCC 43300, **(D)** HYP6, **(E)** 26FS31, **(F)** C2Y, **(G)** 2ZG3, **(H)** YFC18.

Supplementary Figure 2 Clearance rate of geraniol with sub-minimum inhibitory concentrations on preformed biofilms of MSSA standard strains **(A)** ATCC 29213, **(B)** ATCC 25923 and MRSA strains **(C)** ATCC 43300, **(D)** HYP6, **(E)** 26FS31, **(F)** C2Y, **(G)** 2ZG3, **(H)** YFC18.
