# Supplementary material for: Geraniol inhibits biofilm formation of methicillin-resistant Staphylococcus aureus and increase the therapeutic effect of vancomycin in vivo
Source: Front Microbiol. 2022 Sep 6;13:960728. doi: 10.3389/fmicb.2022.960728 (PMC9485828; doi:10.3389/fmicb.2022.960728)
Supplement: Supplementary file 1 [file Table_1.docx]

Supplementary Table S1. The results of MIC expressed as the OD_600nm_ value.

| Concentration of geraniol (μg/mL) | 0 | 16 | 32 | 64 | 128 | 256 | 512 | 1024 |
| --- | --- | --- | --- | --- | --- | --- | --- | --- |
| OD_600nm_ | 2.02 ± 0.03 | 1.86 ± 0.21 | 1.85 ± 0.14 | 1.85 ± 0.04 | 1.87 ± 0.03 | 1.78 ± 0.07 | 0.06 ± 0.03 | 0.06 ± 0.04 |
