# Supplementary material for: Geraniol inhibits biofilm formation of methicillin-resistant Staphylococcus aureus and increase the therapeutic effect of vancomycin in vivo
Source: Front Microbiol. 2022 Sep 6;13:960728. doi: 10.3389/fmicb.2022.960728 (PMC9485828; doi:10.3389/fmicb.2022.960728)
Supplement: Supplementary file 2 [file Table_2.docx]

Supplementary Table S2. The results of growth curve expressed as OD_600nm_.

| Concentration of  geraniol  (μg/mL)  Time (h) | 0 | 16 | 32 | 64 | 128 | 256 | 512 | 1024 |
| --- | --- | --- | --- | --- | --- | --- | --- | --- |
| 0 | 0.30 | 0.30 | 0.30 | 0.30 | 0.30 | 0.30 | 0.30 | 0.30 |
| 1 | 1.00 ± 0.12 | 1.02 ± 0.30 | 1.01 ± 0.35 | 1.08 ± 0.26 | 0.87 ± 0.20 | 0.60 ± 0.28 | 0.25 ± 0.05 | 0.23 ± 0.01 |
| 2 | 1.69 ± 0.04 | 1.34 ± 0.52 | 1.60 ± 0.30 | 1.63 ± 0.26 | 1.60 ± 0.08 | 0.80 ± 0.49 | 0.23 ± 0.06 | 0.23 ± 0.04 |
| 4 | 2.11 ± 0.20 | 1.87 ± 0.40 | 1.94 ± 0.30 | 2.00 ± 0.29 | 1.95 ± 0.22 | 1.63 ± 0.60 | 0.21 ± 0.06 | 0.20 ± 0.04 |
| 6 | 2.42 ± 0.17 | 2.41 ± 0.26 | 2.38 ± 0.29 | 2.36 ± 0.23 | 2.40 ± 0.24 | 1.88 ± 0.61 | 0.20 ± 0.08 | 0.17 ± 0.06 |
| 8 | 2.60 ± 0.16 | 2.42 ± 0.21 | 2.50 ± 0.31 | 2.36 ± 0.20 | 2.43 ± 0.20 | 1.99 ± 0.32 | 0.19 ± 0.09 | 0.17 ± 0.07 |
| 12 | 2.72 ± 0.17 | 2.65 ± 0.26 | 2.60 ± 0.30 | 2.51 ± 0.19 | 2.64 ± 0.16 | 2.48 ± 0.24 | 0.19 ± 0.09 | 0.14 ± 0.09 |
| 24 | 2.84 ± 0.14 | 2.80 ± 0.25 | 2.82 ± 0.25 | 2.81 ± 0.23 | 2.78 ± 0.28 | 2.69 ± 0.30 | 0.17 ± 0.07 | 0.14 ± 0.09 |
