# Supplementary material for: Geraniol inhibits biofilm formation of methicillin-resistant Staphylococcus aureus and increase the therapeutic effect of vancomycin in vivo
Source: Front Microbiol. 2022 Sep 6;13:960728. doi: 10.3389/fmicb.2022.960728 (PMC9485828; doi:10.3389/fmicb.2022.960728)
Supplement: Supplementary file 3 [file Table_3.docx]

| *Staphylococcus aureus* strains | MIC (μg/mL) |
| --- | --- |
| MSSA, ATCC 25923 | 512 |
| MSSA, ATCC 29213 | 512 |
| MRSA, USA300 | 512 |
| MRSA, ATCC 43300 | 512 |
| MRSA, clinical strain HYP6 | 512 |
| MRSA, clinical strain C2Y | 512 |
| MRSA, clinical strain 2ZG3 | 512 |
| MRSA, clinical strain 26FS31 | 512 |
| MRSA, clinical strain YFC18 | 256 |

Supplementary Table S3. Activity of geraniol against several strains of *staphylococcus aureus*. MSSA, methicillin-susceptible *S. aureus*; MRSA, methicillin-resistant *S. aureus*
