# Supplementary figures and images for: Geraniol inhibits biofilm formation of methicillin-resistant Staphylococcus aureus and increase the therapeutic effect of vancomycin in vivo
Source: Front Microbiol. 2022 Sep 6;13:960728. doi: 10.3389/fmicb.2022.960728 (PMC9485828; doi:10.3389/fmicb.2022.960728)

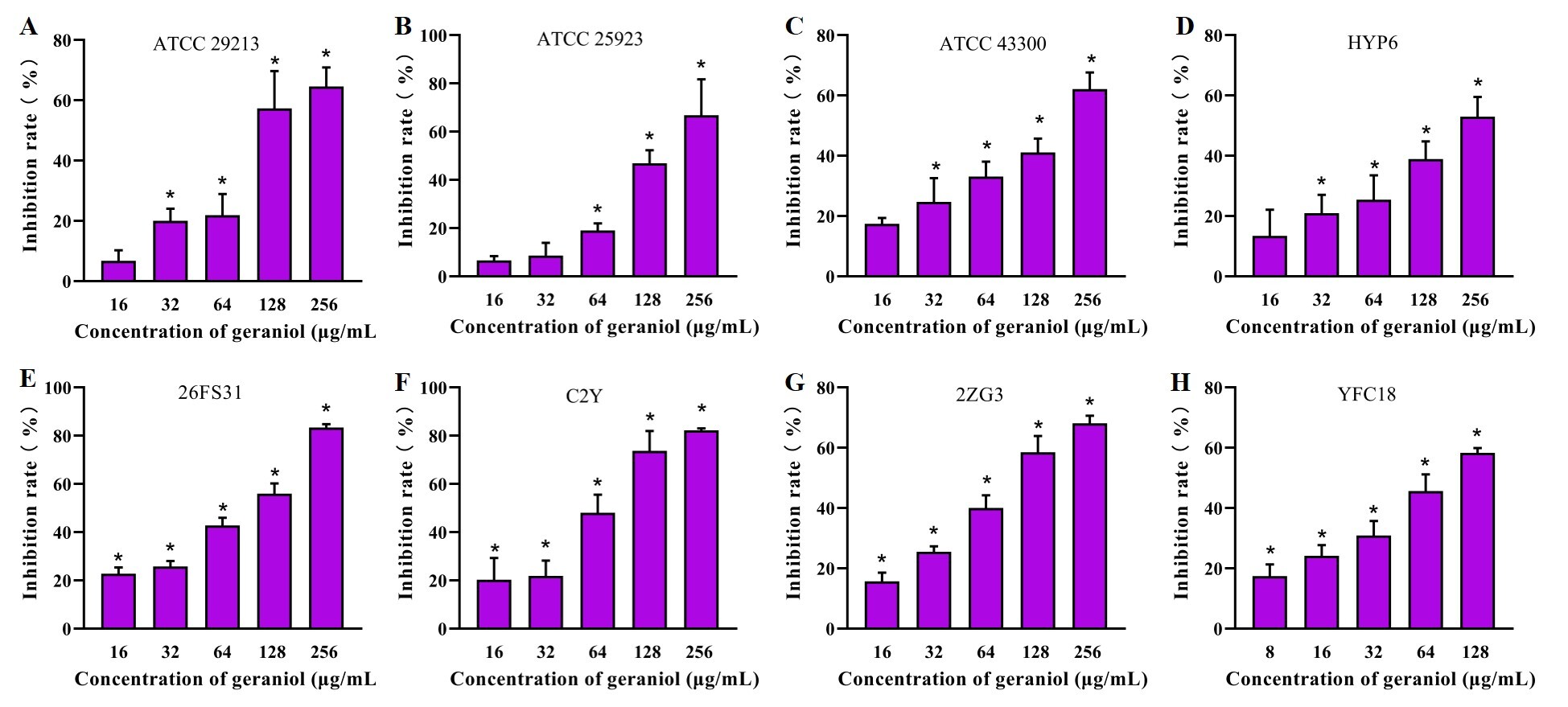

Supplement: Supplementary Figure 1 — Inhibition rate of geraniol with sub-minimum inhibitory concentrations on the formation of MSSA standard strains (A) ATCC 29213, (B) ATCC 25923 and MRSA strains (C) ATCC 43300, (D) HYP6, (E) 26FS31, (F) C2Y, (G) 2ZG3, (H) YFC18. [file Image_1.JPEG]

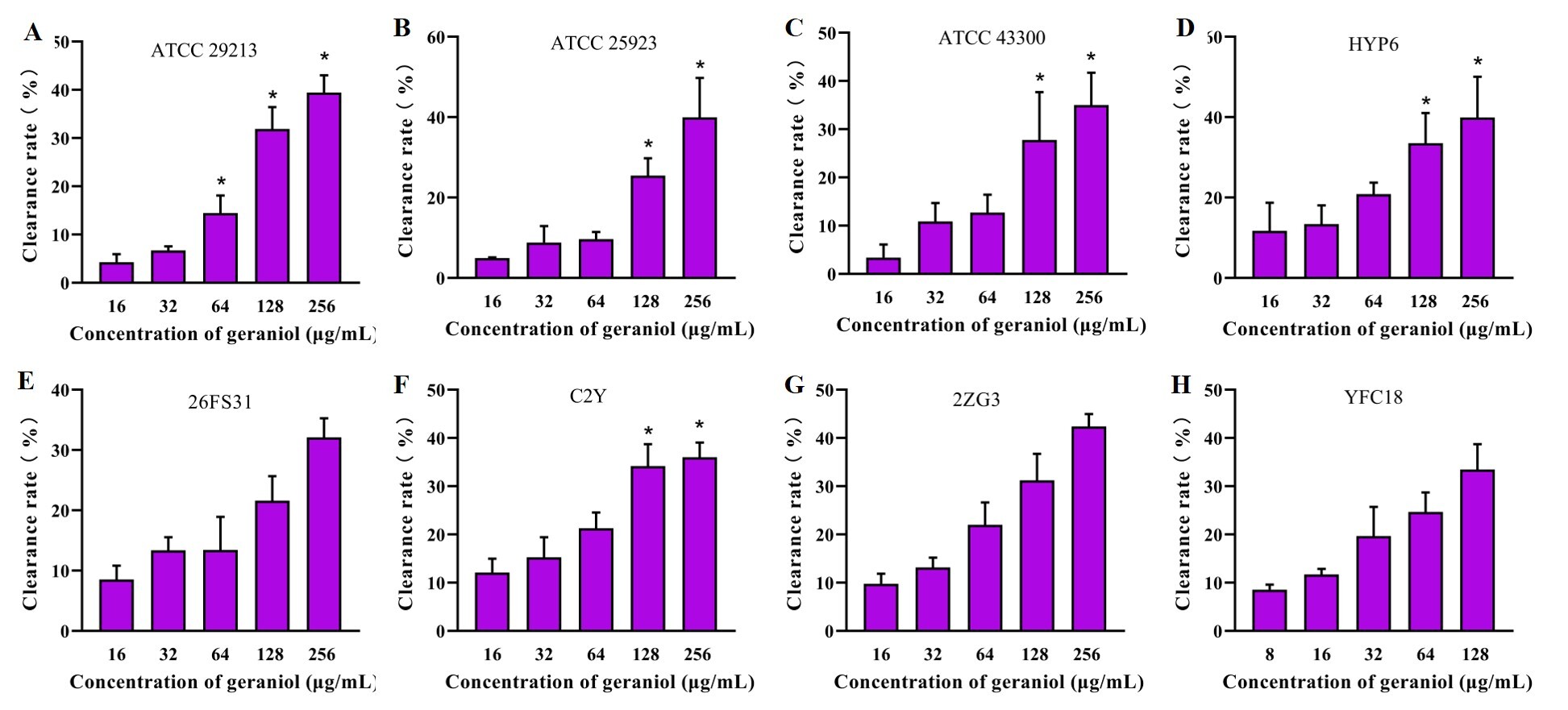

Supplement: Supplementary Figure 2 — Clearance rate of geraniol with sub-minimum inhibitory concentrations on preformed biofilms of MSSA standard strains (A) ATCC 29213, (B) ATCC 25923 and MRSA strains (C) ATCC 43300, (D) HYP6, (E) 26FS31, (F) C2Y, (G) 2ZG3, (H) YFC18. [file Image_2.JPEG]

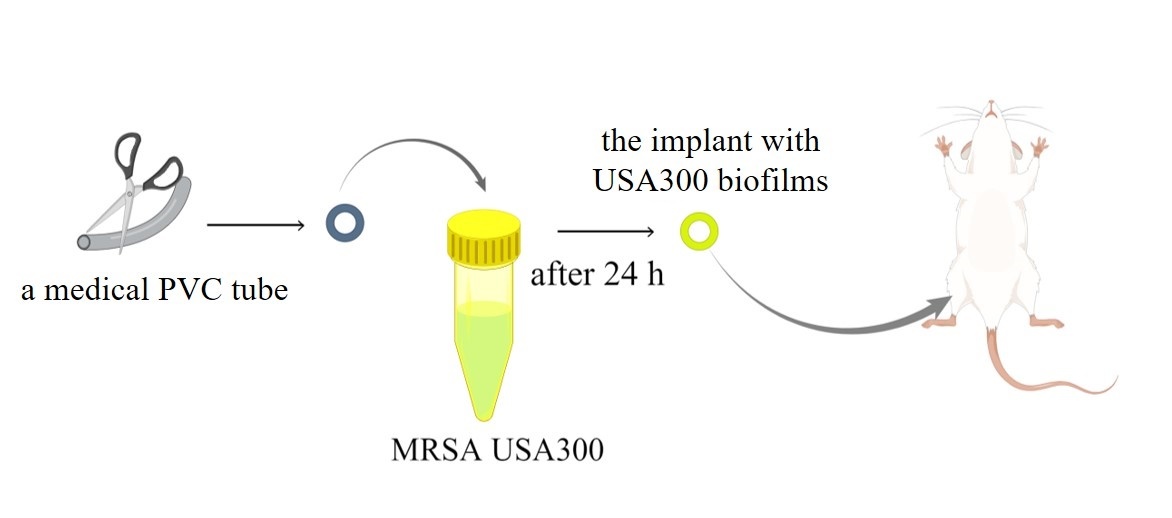

Supplement: Supplementary file 7 [file Image_3.jpg]
